# Herpes Simplex Virus 1 Counteracts Tetherin Restriction via Its Virion Host Shutoff Activity

**DOI:** 10.1128/JVI.02167-13

**Published:** 2013-12

**Authors:** Helen L. Zenner, Rui Mauricio, George Banting, Colin M. Crump

**Affiliations:** Department of Pathology, University of Cambridge, Cambridge, United Kingdoma; Department of Biochemistry, University of Bristol, Bristol, United Kingdomb

## Abstract

The interferon-inducible membrane protein tetherin (Bst-2, or CD317) is an antiviral factor that inhibits enveloped virus release by cross-linking newly formed virus particles to the producing cell. The majority of viruses that are sensitive to tetherin restriction appear to be those that acquire their envelopes at the plasma membrane, although many viruses, including herpesviruses, envelope at intracellular membranes, and the effect of tetherin on such viruses has been less well studied. We investigated the tetherin sensitivity and possible countermeasures of herpes simplex virus 1 (HSV-1). We found that overexpression of tetherin inhibits HSV-1 release and that HSV-1 efficiently depletes tetherin from infected cells. We further show that the virion host shutoff protein (Vhs) is important for depletion of tetherin mRNA and protein and that removal of tetherin compensates for defects in replication and release of a Vhs-null virus. Vhs is known to be important for HSV-1 to evade the innate immune response *in vivo*. Taken together, our data suggest that tetherin has antiviral activity toward HSV-1 and that the removal of tetherin by Vhs is important for the efficient replication and dissemination of HSV-1.

## INTRODUCTION

Tetherin (Bst-2, or CD317) is an interferon (IFN)-inducible membrane glycoprotein that has been shown to have potent antiviral activity by inhibiting the release of many enveloped viruses (1, 2). Tetherin has an unusual topology with a short N-terminal cytoplasmic domain followed by a transmembrane domain, an extracellular loop, and a C-terminal glycosylphosphatidylinositol (GPI) anchor. The extracellular domain of tetherin forms parallel homodimers via coiled-coil interactions and 1 to 3 disulfide bonds (3, 4). Tetherin restricts virus release by embedding one membrane-anchored end in the viral membrane during envelopment, with the other end of tetherin remaining in the host membrane, forming a proteinaceous cross-link between virus particles and the infected cell (4, 5). This traps virions on the surfaces of infected cells and can also result in the endocytosis and lysosomal degradation of trapped virions. The ability of tetherin to detect and target budding viral membranes provides a broad-spectrum antiviral activity against a diverse range of virus families. Recently, tetherin has also been shown to activate NF-κB-dependent gene expression in response to virus particle formation, adding an additional layer to tetherin-mediated antiviral activity (6, 7).

Tetherin is localized both on the plasma membrane and in intracellular compartments such as the *trans*-Golgi network (TGN) and endosomes (8). Current research suggests that the primary site of tetherin's antiviral activity is the cell surface, with the majority of viruses so far having been shown to be restricted by tetherin being those that acquire their envelope by directly budding through the plasma membrane (1, 2). However, many enveloped viruses acquire their membranes by budding at intracellular organelles, and there is less understanding of tetherin-mediated antiviral activity toward these viruses. *Herpesviridae* is one such family, having a complex assembly pathway culminating in the final envelopment event occurring at membranes derived from TGN and/or endosomal compartments (9–11). So far, the effect of tetherin on two herpesviruses has been investigated with conflicting observations. Two independent studies have demonstrated that tetherin expression inhibits the release of Kaposi's sarcoma-associated herpesvirus (KSHV; a gammaherpesvirus) (12, 13). However, a further study has demonstrated that tetherin expression does not restrict human cytomegalovirus (HCMV; a betaherpesvirus) but conversely appears to enhance viral entry (14). Thus far, no studies have been published on the effect of tetherin on the replication of alphaherpesviruses.

We were interested in whether tetherin could function as a host defense factor against alphaherpesviruses such as herpes simplex virus 1 (HSV-1). Like many herpesviruses, HSV-1 is predominantly cell associated and can efficiently spread directly from cell to cell, making it uncertain whether simply tethering virions to the cell surface would have a dramatic effect on HSV-1 dissemination. Indeed, tetherin does not appear to prevent direct cell-to-cell spread of HIV-1 infection via virological synapses (15). However, tetherin's antiviral function is likely to be more than just a physical method of restricting virus release from the cell surface, with internalization and degradation of virions as well as activation of proinflammatory gene expression via NF-κB potentially contributing to tetherin-mediated defense against virus infection (6, 7). Studies with tetherin knockout mice have demonstrated that tetherin is important for the restriction of murine leukemia virus replication and disease *in vivo*, confirming its role as a bona fide antiviral factor (16). Interestingly, studies with another knockout mouse have shown that tetherin deletion inhibits alpha interferon (IFN-α) secretion in response to HSV-1 or mouse cytomegalovirus infection (17), suggesting a role for tetherin in sensing/responding to herpesviruses *in vivo*.

Viruses have evolved a variety of countermeasures against tetherin activity, the majority of which cause the removal of tetherin from the cell surface followed by either sequestration in intracellular compartments or degradation in the lysosomes (1, 2). However, simply causing the internalization of tetherin may not block potential antiviral activity toward herpesviruses, because these viruses assemble at compartments in direct contact with endocytosed material (TGN/endosomes). KSHV, so far the only known herpesvirus to be restricted by tetherin, utilizes its immune evasion protein, K5, to ubiquitinate tetherin and cause its ESCRT-mediated lysosomal degradation (13, 18).

An important HSV-1 gene for evading IFN-responses is UL41, which encodes Vhs (virion host shutoff protein) (19). Vhs is an endoribonuclease that is packaged into the tegument of HSV-1 and causes the destabilization of a broad spectrum of mRNA molecules (20). Despite a wide range of potential targets of Vhs activity, it appears that one of the major roles of Vhs *in vivo* is to antagonize both innate and adaptive immune responses: in addition to inhibiting IFN responses, Vhs can modulate antigen presentation, dendritic cell maturation, and cytokine/chemokine responses (21–23). This is reflected in the fact that Vhs-null viruses replicate efficiently in cell culture but are highly attenuated *in vivo* (24–26). Furthermore, the replication of Vhs-null HSV-1 can be partially restored in STAT1 knockout mice, suggesting that IFN responses cause at least some of the reduced pathogenesis of Vhs-null viruses (27).

In this work, we investigated two main questions: (i) does tetherin restrict HSV-1 release from infected cells? and (ii) is tetherin an important target of Vhs activity? Our data demonstrate the following: tetherin expression inhibits the release of HSV-1, wild-type HSV-1 efficiently blocks tetherin expression, and Vhs activity is important for HSV-1 to evade tetherin restriction. Our data thus uncover a novel method of tetherin antagonism by viruses: targeting tetherin mRNA for destruction.

## MATERIALS AND METHODS

### Cells.

All cells were maintained in Dulbecco's modified Eagle medium (DMEM) supplemented with 10% (vol/vol) fetal calf serum (FCS), 100 U/ml penicillin G, 0.1 mg/ml streptomycin, and 2 mM glutamine. Caco-2 cells stably expressing short-hairpin RNA (shRNA) were maintained in medium supplemented with 250 μg/ml hygromycin. HS30 cells were maintained in medium supplemented with 200 μg/ml G418.

### Viruses.

All recombinant viruses were generated using a two-step Red recombination technique (28) in Escherichia coli strain GS1783 (from G. Smith, Northwestern University) harboring the BAC-cloned HSV-1 genome (strain KOS; from D.A. Leib, Dartmouth Medical School [29]). Firstly, HSV-1ΔVhs was constructed by replacing codons 46 to 50 of UL41 with 3 tandem in-frame stop codons using primers COL428 (CGTGGACCTGTGGAACGTCATGTACACGTTGGTGGTCAAATAATAGTGAATTCCCCAGTTACGACCGCGAGGAGGATGACGACGATAAGTAGGG) and COL429 (GAGGCAGTGTAGCGTAATGGCCTCGCGGTCGTAACTGGGGAATTCACTATTATTTGACCACCAACGTGTACACAACCAATTAACCAATTCTGATTAG). Then wild-type HSV-1 (HSV-1wt) and HSV-1ΔVhs BAC DNAs were used as templates to insert enhanced yellow fluorescent protein [EYFP(A206K)] in frame after codon 490 of UL48 to create viruses expressing a VP16-EYFP fusion protein using primers SS09 and SS10, and expression of VP16-EYFP was verified by immunofluorescence assay and Western blotting as previously described (30).

### HSV-1 release assay.

The cell lines HeLa, Vero, Caco-2, and Caco-2–tetherin shRNA were inoculated with HSV-1wt or HSV-1ΔVhs at 5 PFU/cell for 1 h followed by an acid wash (40 mM citric acid, 135 mM NaCl, 10 mM KCl [pH 3.0]) to inactivate residual input virus. At 16 h postinfection, culture medium was carefully removed and centrifuged twice at 5,000 rpm for 5 min in a microcentrifuge to remove cellular debris. Cells were scraped into medium and subjected to sonication or freeze-thawing to liberate cell-associated virus. Infectious virus present in media and cell samples was assayed by plaque assay on Vero cell monolayers, and the proportion of HSV-1 release was determined by dividing supernatant virus titers by cell-associated plus supernatant titers. Statistical analysis was performed using Student's *t* test.

### Complementation assay.

COS-7 cells were transfected with 0.5 μg of a plasmid expressing the HSV-1 UL36 gene (VP1/2) with or without 0.03 μg of pCR3.1-HA-Tetherin or pCR3.1-HA-TetherinΔGPI using Trans-IT LT1 following the manufacturer's instructions (Mirus Bio LLC). An equivalent amount of DNA was maintained in all samples by the inclusion of empty pCR3.1 vector. At 24 h posttransfection, cells were infected with HSV-1ΔUL36 at 5 PFU/cell for 1 h followed by an acid wash to inactivate input virus. Supernatant and cell-associated samples were collected as described above and assayed for plaque formation on a UL36 (VP1/2) complementing cell line (HS30).

### HSV-1 growth curves.

HeLa, Vero, Caco-2, and Caco-2–tetherin shRNA (Caco-2 expressing a tetherin shRNA) cell lines were inoculated with HSV-1wt or HSV-1ΔVhs at 5 PFU/cell (single-cycle growth curves) or 0.01 PFU/cell (multicycle growth curves) for 1 h at 37°C. The residual virus was inactivated by an acid wash for 1 min at room temperature and/or three washes with phosphate-buffered saline (PBS). At various times postinfection, cells were harvested and lysed by sonication or freeze-thawing. Virus yield was determined by plaque assay on Vero cells.

### Reverse transcription-quantitative PCR (RT-qPCR).

HeLa cells were infected with HSV-1wt or HSV-1ΔVhs at 5 PFU/cell or mock infected. At various times postinfection, total RNA was prepared using TRIzol reagent according to manufacturer's instructions (Invitrogen). cDNA was synthesized using SuperScript III reverse transcriptase (Invitrogen) with random hexamers. Real-time PCR was performed using a Rotorgene (Corbett Research) in triplicate for each sample. Primers and probes were designed by Tib-MolBiol (Berlin). A primer set for the detection of tetherin mRNA was based on the following sequences, which spanned an exon-exon junction: forward primer, TGATGGCCCTAATGGCTTCC; reverse primer, AGACCTGGTTTTCTCTTCTCAGTCG; TaqMan probe, 6FAM-CCTCAAGCTCCTCCACTTTCTTTTGTCCTT-BBQ. For normalization, a primer set for the detection of 18S rRNA was used based on the following sequences: forward primer, CGGCTACCACATCCAAGGAA; reverse primer, GCTGGAATTACCGCGGCT; TaqMan probe, 6FAM-CGCAAATTACCCACTCCCGACCC-TMR. Results from the real-time PCRs were quantified as copy number per sample using the Rotorgene software from standard curves that were generated using known amounts of plasmids containing each of the relevant DNA regions. The data set for tetherin was divided by the mean of the triplicate 18S rRNA data set from the same DNA sample to obtain estimates of the relative number tetherin mRNA copies present per cell. Data for each time point were normalized to those for mock-treated controls.

### Western blotting.

Cells were harvested, pelleted, and lysed in 0.1 ml of lysis buffer (50 mM Tris-HCl [pH 7.4], 150 mM NaCl, 1% sodium deoxycholate, 1% Triton X-100) supplemented with protease inhibitor cocktail (Roche) for 20 min on ice followed by centrifugation at 17,000 × *g* for 10 min at 4°C. Samples were boiled with SDS-PAGE sample buffer for 5 min, separated on polyacrylamide gels, and electrophoretically transferred to nitrocellulose membranes. Membranes were blocked and incubated with primary antibodies against tetherin (11721; NIH AIDS Reagent Program), VP16 (ab110226; Abcam), VP1/2 (CB4 [30]), a hemagglutinin (HA) tag (HA.11; Covance), or actin (AC-40; Sigma), followed by horseradish peroxidase (HRP)-, IRDye680-, or IRDye800-conjugated secondary antibodies, and developed by enhanced chemiluminescence (ECL) or by using an Odyssey infrared imaging system (LI-COR). Band intensities were quantified using ImageJ, and tetherin signals were normalized to actin loading controls.

### Immunofluorescence.

HeLa cells grown on 13-mm coverslips were infected at a multiplicity of infection (MOI) of 3 PFU/cell for 18 h. The cells were then fixed in 3% formaldehyde in PBS for 15 min, permeabilized, and quenched using 0.2% saponin and 50 mM NH_4_Cl. Blocking and antibody dilution were carried out in PBS with 0.2% gelatin, 0.02% saponin, and 0.02% sodium azide. Samples were incubated with primary antibodies against tetherin (mouse polyclonal; ab88523; Abcam) and HSV-1 glycoprotein M (rabbit polyclonal) followed by secondary antibodies conjugated to Alexa 488 and 568 (Molecular Probes), and coverslips were mounted in ProLong Gold antifade mounting medium (Invitrogen). Images were acquired using an Olympus IX70 fluorescence microscope equipped with a 60× oil immersion lens. Images were captured using a Reitga 2000R charge-coupled device camera and Q capture Pro (Media Cybernetics) and processed using Adobe Photoshop.

### Plaque spread assay.

Caco-2 and Caco-2–tetherin shRNA cells were grown until they formed confluent monolayers and were then inoculated with HSV-1wt-VP16EYFP or HSV-1ΔVhs-VP16EYFP at ∼1,000 PFU per well for 1 h at 37°C. Cells were overlaid with DMEM supplemented with 2% FCS, 100 U/ml penicillin G, 0.1 mg/ml streptomycin, 2 mM glutamine, and 0.6% carboxymethylcellulose. At 3 days postinfection, cells were fixed and stained with DAPI (4′,6-diamidino-2-phenylindole), and images of fluorescent plaques were acquired using an Olympus IX81 fluorescence microscope equipped with a 10× lens. Plaque diameters were measured using Image Pro Plus (Media Cybernetics).

## RESULTS

**Fig 1:**
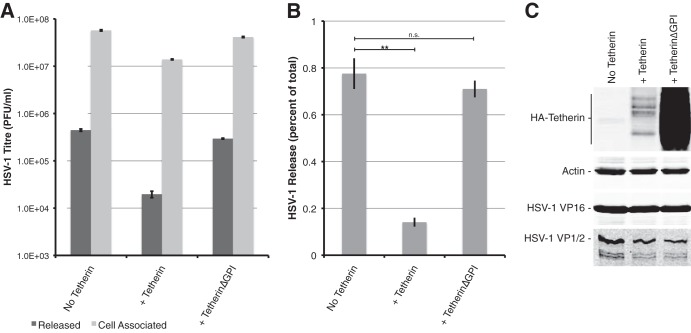
Tetherin expression inhibits HSV-1 release. (A) COS7 cells were transfected with control, tetherin, or tetherinΔGPI expression plasmids together with a plasmid expressing the HSV-1 UL36 gene (VP1/2). Cells were infected with HSV-1ΔUL36, and titers of infectious virus both in the supernatant (dark bars) and associated with cells (light bars) were established 16 h postinfection. Error bars represent standard errors of the means for duplicate samples. (B) HSV-1 release was calculated as percentage of total infectious virus for each sample. Error bars represent standard errors of the means. ***, *P* < 0.005; **, *P* < 0.05; *, *P* < 0.1; n.s., *P* > 0.1. (C) Cell extracts were analyzed by Western blotting with antibodies specific for the HA tag, actin, VP16, and VP1/2.

To examine the effect of tetherin expression on HSV-1 release, we determined the amount of infectious virus released into culture supernatant compared to the amount that remained cell associated using COS-7 cells expressing HA-tagged versions of wild-type human tetherin or human tetherin lacking the C-terminal GPI anchor, which is unable to restrict virus release. For these experiments, we utilized a transcomplementation assay, previously described (31), where cells are cotransfected with a plasmid expressing the essential HSV-1 protein VP1/2 and then infected with HSV-1 lacking the VP1/2 gene (UL36). This results in the assembly of infectious virus occurring exclusively in transfected cells. The expression of HA-tagged wild-type tetherin caused a modest reduction in cell-associated virus titers (4.1-fold compared to the no-tetherin control) but a greater reduction in released virus titers (22.6-fold compared to the no-tetherin control) (Fig. 1A). Calculation of relative virus release as a percentage of total infectivity demonstrated a significant 5.5-fold inhibition of HSV-1 release caused by tetherin overexpression, whereas there was no significant effect of tetherinΔGPI on HSV-1 release (Fig. 1B). Therefore, as with KSHV, tetherin can specifically restrict HSV-1 secretion. The reason for the reduction in cell-associated infectivity, which was consistently observed over several experiments, is currently unclear, but it could potentially be due to tetherin-mediated internalization and degradation of tethered virions. It is unlikely to be due to effects on cell viability, viral entry, or gene expression; expression of tetherin or tetherinΔGPI had little effect on the levels of cellular actin and viral proteins such as VP16 or on the expression of VP1/2 from the transcomplementing plasmid (Fig. 1C). The expression levels of tetherin lacking the GPI anchor were consistently much higher than those of full-length tetherin, suggesting that the GPI anchor may be important for the normal turnover of tetherin protein.

We next wanted to address whether HSV-1 encodes mechanisms to antagonize endogenous tetherin. HSV-1 expresses an efficient host shutoff activity, primarily due to Vhs, a potent mRNA-targeted endoribonuclease (19). While Vhs has been shown to be dispensable for virus replication in cell culture lines, animal models have demonstrated that Vhs is important for antagonizing interferon-induced antiviral activities (24, 25). Given that tetherin has been shown to be a key effector of the interferon-induced antiviral activity *in vivo* (16), we investigated whether Vhs could be an important factor to combat tetherin activity.

**Fig 2:**
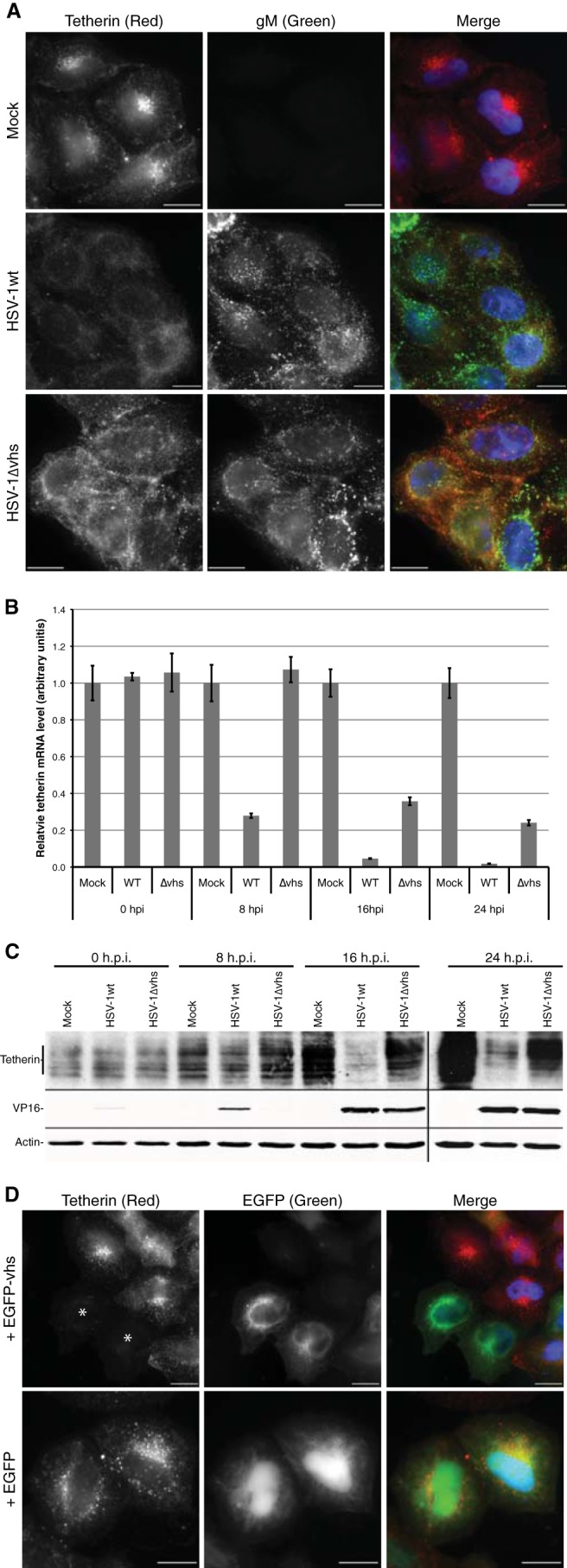
HSV-1 Vhs inhibits tetherin expression. HeLa cells were either mock infected or infected with HSV-1wt or HSV-1ΔVhs. (A) Cells were fixed and stained with antibodies to tetherin (red) and HSV-1 gM (green) at 16 h postinfection and analyzed by immunofluorescence. Alternatively, cells were harvested at 0, 8, 16, and 24 h postinfection, and then relative tetherin mRNA levels were analyzed by RT-qPCR (B) and protein expression by Western blotting (C). (D) HeLa cells were transfected with plasmids expressing either GFP or GFP-Vhs (green). At 24 h posttransfection, the cells were fixed and labeled with anti-tetherin (red). The asterisks indicate GFP-Vhs-positive cells. Bars, 20 μm.

First, using a BAC-cloned HSV-1 genome, we generated a Vhs-null (ΔVhs) virus by replacing codons 46 to 50 of UL41 with a cassette containing 3 consecutive in-frame stop codons followed by a frameshift. HeLa cells, which naturally express tetherin (32), were infected with HSV-1wt or HSV-1ΔVhs, and endogenous tetherin levels were analyzed at 18 h postinfection (hpi) by immunofluorescence (Fig. 2A). Mock-infected cells demonstrated a strong signal for tetherin localized to a perinuclear compartment, reminiscent of the TGN, as well as cell surface staining. In cells infected with HSV-1wt, endogenous tetherin was virtually undetectable, with only background signal levels being evident. However, in cells infected with HSV-1ΔVhs, strong tetherin signals were observed, although with a different distribution than in uninfected cells: tetherin appeared to be more prevalent at the cell surface as well as being present in a punctate pattern throughout the cytoplasm. This suggests a partial relocalization of tetherin to the plasma membrane in HSV-1ΔVhs-infected cells, where secreted virions would be expected to accumulate. Loss of perinuclear staining may be due to fragmentation of the Golgi complex that is induced by HSV-1 at late times postinfection (33).

Tetherin mRNA and protein levels were analyzed at various times postinfection by RT-qPCR and Western blotting, respectively, in HeLa cells infected with HSV-1wt and HSV-1ΔVhs. Infection by wild-type HSV-1 led to a rapid decrease in tetherin mRNA, with a >3.5-fold decrease by 8 h and a >20-fold decrease by 16 h postinfection (Fig. 2B). Conversely, infection by HSV-1ΔVhs caused no change in tetherin mRNA levels by 8 h and a <3-fold decrease by 16 h postinfection. This suggests that Vhs mediates the specific degradation of tetherin mRNA at earlier times postinfection, while in the absence of Vhs expression, other viral factors, or possibly the general cytopathic effect of HSV-1, cause a more modest decline in tetherin mRNA at later times postinfection. Quantification of protein levels demonstrated a slight reduction in tetherin in HSV-1wt-infected cells by 8 h (1.2-fold) and a large reduction by 16 h postinfection (3.4-fold) (Fig. 2C). No reduction in tetherin protein levels was observed in cells infected with HSV-1ΔVhs by 8 h postinfection. A slightly lower level of tetherin protein was observed in HSV-1ΔVhs-infected than uninfected cells at 16 and 24 h (1.3- to 1.4-fold), which could be due to other HSV-1 factors affecting tetherin expression or turnover but may also be partially due to the continued cell division and growth of uninfected cells. A lower expression level of the major tegument protein VP16 was observed in HSV-1ΔVhs-infected than in HSV-1wt-infected cells at 8 h, although similar levels were apparent at 16 and 24 h postinfection. Vhs has previously been reported to be important for the efficient expression of late viral genes in HeLa cells (34).

HSV-1 Vhs is known to target many viral and cellular mRNAs for destruction. To address whether inhibition of tetherin expression is due directly to Vhs rather than indirectly via another HSV-1 gene that is itself regulated by Vhs activity, tetherin expression was analyzed in HeLa cells transfected with plasmids expressing EGFP-Vhs or EGFP only. Fluorescence microscopy demonstrated EGFP-Vhs expressing cells lacked any detectable tetherin expression, while control levels of tetherin were observed in EGFP expressing cells, Therefore, tetherin expression appears to be directly inhibited by Vhs activity (Fig. 2D).

**Fig 3:**
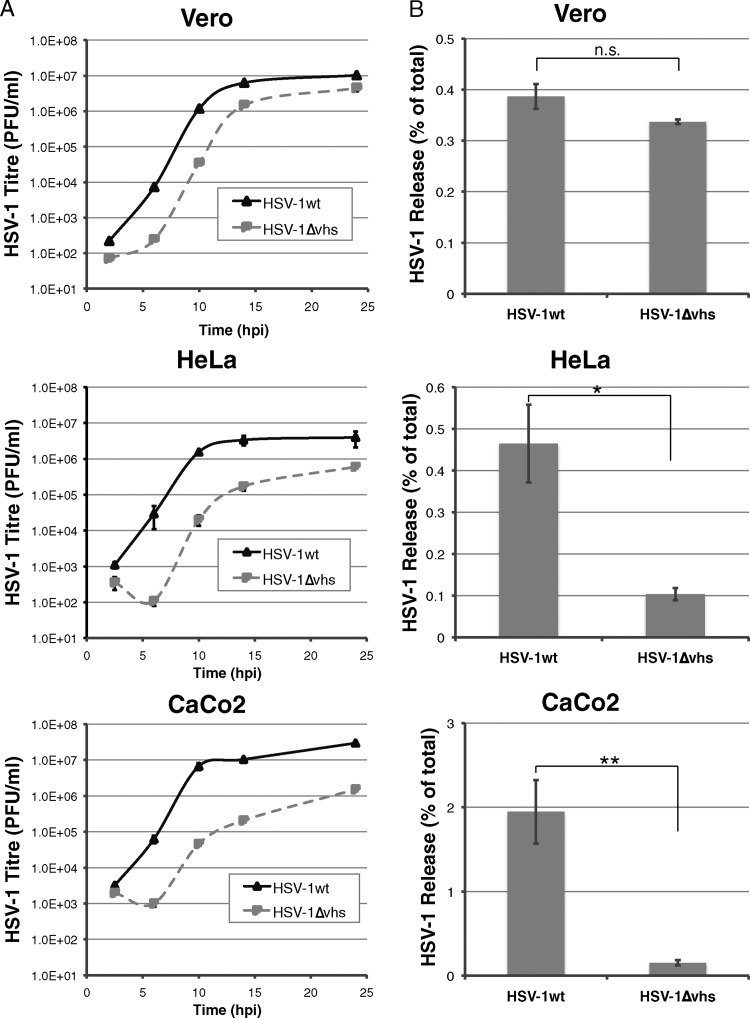
HSV-1ΔVhs is released poorly from tetherin-expressing cell lines. (A) Vero, HeLa, and Caco-2 cells were infected with either HSV-1wt (solid line) or HSV-1ΔVhs (dashed line) at 5 PFU/cell. Total infectious virus yields at the indicated time points were determined by plaque assay on Vero cells. Error bars represent standard errors of the means for duplicate samples. (B) At 16 h postinfection, the amounts of infectious virus present both in the supernatant and associated with the cell were determined by plaque assay on Vero cells, and the proportion of released virus was determined as a percentage of total infectious virus. The data are averages from at least 2 independent experiments, performed with triplicate samples for each condition. Error bars represent standard errors of the means. ***, *P* < 0.005; **, *P* < 0.05; *, *P* < 0.1; n.s., *P* > 0.1.

We next asked whether the loss of Vhs expression affected the release of HSV-1 particles from infected cells. To investigate this, we utilized three different cell lines: Vero, a primate cell line that lacks an interferon response and does not express detectable levels of tetherin (35), and two human cell lines, HeLa and Caco-2, that constitutively express tetherin (32, 36). Single-cycle growth curve analysis of HSV-1 replication in these cell lines demonstrated that HSV-1ΔVhs replicated more slowly in all cell lines, but the difference between HSV-1ΔVhs and HSV-1wt was more pronounced in both HeLa and Caco-2 cells (Fig. 3A). A greater attenuation of HSV-1ΔVhs replication in certain human cell lines, including HeLa cells, has been previously reported, and this was correlated with the requirement of Vhs for efficient viral late-gene expression in these cell types (34). Analysis of the proportion of HSV-1 released into the culture media demonstrated no significant difference between the wild-type and ΔVhs viruses in infected Vero cells, whereas a significant inhibition of virus release was observed in both HeLa cells (4.5-fold) and Caco-2 cells (12.6-fold) for HSV-1ΔVhs (Fig. 3B). These data suggest that loss of Vhs expression causes a specific defect in HSV-1 release from tetherin-expressing cells.

**Fig 4:**
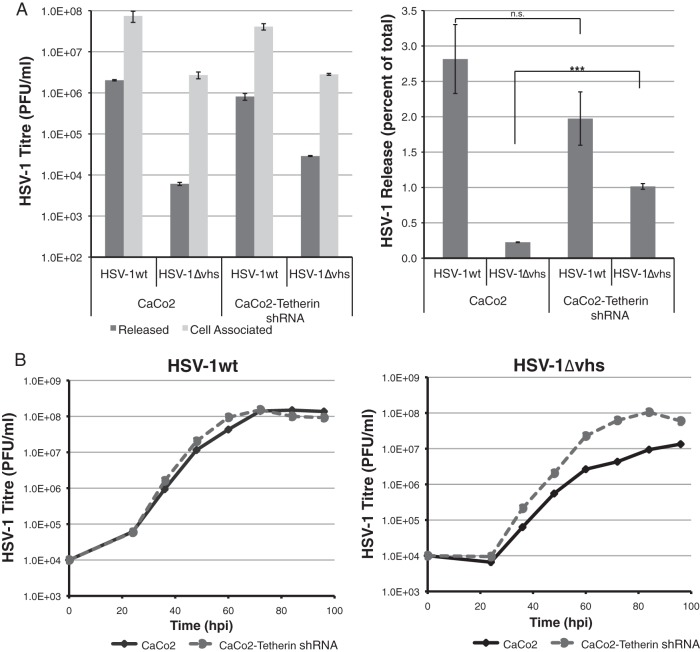
Release and multistep replication of HSV-1ΔVhs is inhibited in a tetherin-dependent manner. Caco-2 cells and Caco-2–tetherin shRNA cells were infected with HSV-1wt or HSV-1ΔVhs. (A) Infectious-HSV-1 titers both in supernatant (dark bars) and associated with cells (light bars) were established 16 h postinfection. HSV-1 release was calculated as percent of total infectious virus for each sample. Error bars represent standard errors of the means. ***, *P* < 0.005; **, *P* < 0.05; *, *P* < 0.1; n.s., *P* > 0.1. (B) Caco-2 cells (solid lines) and Caco-2–tetherin shRNA cells (dashed lines) were infected at a low MOI (0.01 PFU/cell) with HSV-1wt and HSV-1ΔVhs. Total infectious-virus yields at the indicated time points were determined by plaque assay on Vero cells. Error bars represent standard errors of the means for triplicate samples.

To investigate whether the observed inhibition of HSV-1ΔVhs release was caused by tetherin expression, we utilized a Caco-2 cell line depleted of tetherin, due to the stable expression of a tetherin-specific shRNA (36). Analysis of cell-associated and released virus fractions from control and tetherin-depleted Caco-2 cells showed that there was no significant difference in the release of wild-type HSV-1 in the presence or absence of tetherin. However, HSV-1ΔVhs demonstrated a significant increase (4.5-fold) in virus release from tetherin-depleted Caco-2 cells compared to control Caco-2 cells (Fig. 4A). These data indicate that tetherin expression is an important factor for the inhibition of HSV-1ΔVhs release from infected cells.

The restriction of HSV-1 release by tetherin would be expected to inhibit the spread of virus to uninfected cells. To investigate this, we performed multicycle growth curve analysis of HSV-1wt and HSV-1ΔVhs by infecting subconfluent cultures of control and tetherin-depleted Caco-2 cells at a multiplicity of infection of 0.01 PFU/cell and determined infectious-virus production at various time points over 4 days. HSV-1wt replicated with virtually identical kinetics in both cell types, indicating that the expression of tetherin does not affect the replication or spread of wild-type virus (Fig. 4B, left). HSV-1ΔVhs, however, demonstrated a more rapid and efficient replication in tetherin knockdown cells than control cells (Fig. 4B, right). These data support a tetherin-specific inhibition of HSV-1 spread that is normally overcome by Vhs function.

**Fig 5:**
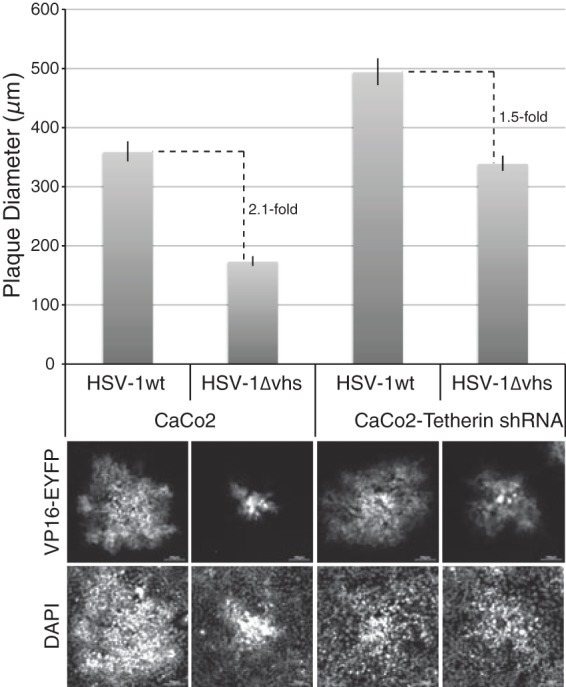
Plaque formation of HSV-1ΔVhs is inhibited in a tetherin-dependent manner. Caco-2 cells and Caco-2–tetherin shRNA cells were infected with HSV-1wt-VP16EYFP or HSV-1ΔVhs-VP16EYFP at ∼1,000 PFU/well in 6-well dishes. At 3 days postinfection, images of fluorescent plaques were taken using a 10× objective lens. (Top) Relative diameters of >40 plaques for each condition were determined using Image Pro Plus (Media Cybernetics), and mean diameters ± standard errors are shown. (Bottom) Representative images of fluorescent plaques are shown. Bars, 100 μm.

To further investigate the effect of tetherin and Vhs on HSV-1 cell-to-cell spread, we monitored plaque formation in confluent cell monolayers. Initial experiments demonstrated that HSV-1wt formed very small plaques on Caco-2 cells, and monitoring cytopathic effect by staining fixed cell monolayers did not allow reliable measurements of plaque diameter. Therefore, to facilitate these assays, we generated recombinant viruses expressing EYFP-tagged VP16, in the background of HSV-1wt and HSV-1ΔVhs, and measured the diameters of fluorescent plaques formed by these viruses in control and tetherin-depleted Caco-2 cell monolayers. The overall size of plaques was found to vary depending on cell density, making it difficult to accurately compare plaque size between Caco-2 and Caco-2–tetherin shRNA cell lines. However, the relative plaque sizes of HSV-1wt and HSV-1ΔVhs could be reliably compared for each individual cell line, and the difference (fold) between the two viruses could then be determined. Analysis of the relative plaque size demonstrated that the absence of Vhs caused a larger reduction in plaque size compared to wild-type virus in Caco-2 cells than it did in tetherin-depleted Caco-2 cells (Fig. 5). Seven complete data sets of plaque measurements for the two viruses in both cell lines from 3 independent experiments, where cells were seeded at various densities, were collected and analyzed. These data showed a highly significant difference (*P* = 0.00033) in the reduction of plaque diameter caused by the deletion of Vhs between Caco-2 cells (2.123- ± 0.096-fold) and tetherin-depleted Caco-2 cells (1.588- ± 0.070-fold). These data suggest that tetherin at least partially inhibits the direct cell-to-cell spread of HSV-1 during plaque formation when Vhs is absent.

Taken together, our data support an important role for Vhs in overcoming antiviral functions in infected cells and, furthermore, show that tetherin is an important target of Vhs. Tetherin thus appears to be a potent restriction factor that can inhibit HSV-1 release and dissemination, and Vhs is at least one mechanism employed by HSV-1 to counteract tetherin.

## DISCUSSION

This study demonstrates that HSV-1 is sensitive to tetherin's antiviral activity and that the viral protein Vhs is one mechanism by which HSV-1 can overcome tetherin restriction. Vhs is a well-established virulence factor for HSV-1 that is thought to have a major role in inhibiting innate immune responses, although it is still unclear which antiviral host factors are genuine targets of Vhs (21–24). Tetherin appears to be an important antiviral factor in mammalian hosts, as highlighted by the numerous and diverse mechanisms viruses have evolved to antagonize tetherin function (1, 2). Our data now provide evidence that tetherin is one key target of HSV-1 Vhs activity and furthermore highlights a new method that is available to viruses to overcome tetherin activity: inducing tetherin mRNA degradation. To date, all tetherin antagonists have been shown to inhibit tetherin function by targeting the protein via mechanisms such as removal from the plasma membrane and/or inducing tetherin degradation (1, 2). To our knowledge, these studies are the first to show a virus directly targeting tetherin mRNA.

Tetherin has a well-established antiviral activity against many enveloped viruses that acquire their envelopes by budding at the plasma membrane (e.g., retroviruses). However, the effect of tetherin on viruses that acquire their envelope by budding into the lumens of intracellular compartments is less well understood. For tetherin to cross-link such viruses to cellular membranes, it must be localized to intracellular virus assembly sites, and different viruses use a variety of secretory and endocytic compartments for their envelopment. Previous studies have shown a moderate inhibition of hepatitis C virus release by tetherin, suggesting possible tetherin activity at the endoplasmic reticulum, where this flavivirus assembles (37). However, tetherin expression did not inhibit the replication or spread of Rift Valley fever virus, a bunyavirus that assembles at the Golgi, or cowpox virus, a poxvirus that likely acquires its outer membrane at the TGN or endosomal membranes (38). This study did not address whether Rift Valley fever or cowpox viruses can antagonize tetherin function, and so it is possible that tetherin in the absence of the relevant viral factor would restrict these viruses. The data on herpesviruses, which assemble at TGN or endosomal membranes, are also unclear, with evidence for tetherin restriction of KSHV but a surprising enhancement of HCMV entry by tetherin (12–14). Our data support the notion that tetherin does indeed have activity against herpesviruses, at least for the alpha and gamma subfamilies, and that this virus family has evolved at least two independent methods of removing tetherin: Vhs-mediated mRNA degradation and K5-mediated protein degradation.

If tetherin restricts different families of intracellularly assembling viruses, one question would be that of how tetherin is targeted/localized to a diverse range of viral assembly sites. Tetherin is known to associate with membrane microdomains (39, 40), and it is conceivable that partitioning of lipids during the membrane deformation of virus budding could be the route for tetherin insertion into virions. Tetherin can access most secretory and endocytic compartments during normal trafficking within the cell and so has the potential to associate with all known sites of virus envelopment. However, if tetherin has a generic activity toward membrane-budding profiles of enveloped viruses, it is also possible that tetherin could restrict exosomes and the intraluminal vesicles (ILV) of multivesicular endosomes, which have the same topology of budding as enveloped viruses. Whether there is any effect on ILV formation or exosome release in IFN-treated or tetherin-expressing cells is currently unknown.

Vhs is a broad-specificity endoribonuclease *in vitro* that cleaves single-stranded RNA at the 3′ side of U and C residues (20). *In vivo*, however, Vhs is specifically targeted to ribosome-associated mRNA, most likely via interaction with the cap-binding initiation factor complex eIF4F, leading to mRNA cleavage in regions of translation initiation (41, 42). In response to viral infection, various sensors induce signaling events that enable the infected cell to establish an antiviral state and to also release interferon and other cytokines to activate surrounding cells (43). Given that much of the cellular response entails *de novo* gene expression, the ability to rapidly inhibit the translation of cellular proteins is a powerful strategy for viruses to suppress the innate immune response and establish an infection. Vhs is a component of the tegument layer in the virion and so is delivered to the cytoplasm during virus entry (44). Therefore, Vhs is ideally placed to rapidly shut down the translation of cellular mRNAs that occurs in response to infection.

While Vhs has undoubtedly important roles in the inhibition of innate immune responses, it is also likely to have other functions and has recently been shown to enhance translation of late viral genes (34). Indeed, in STAT1 and IFN-αβγ receptor knockout mice, HSV-1ΔVhs replication is increased but not rescued to wild-type levels, suggesting that HSV-1 replication requires Vhs activity for more than just evading type I and II IFN responses (34). Interestingly, tetherin expression is also induced by the type III IFN-λ3, and by IRF7 independently of IFN signaling, (45, 46). This suggests that tetherin can still be expressed in response to virus infection even in the absence of IFN-α/β and -γ receptors. It will be interesting to investigate HSV-1ΔVhs pathogenesis in mice lacking tetherin expression in addition to STAT1 or IFN-αβγ receptors to determine whether tetherin contributes to the reduced pathogenesis of HSV-1ΔVhs that still occurs even when type I and II IFN responses are absent. It will also be interesting to investigate whether HSV-1ΔVhs release and spread can be rescued by an alternative tetherin antagonist, such as HIV-1 Vpu or KSHV K5.

Interestingly, as well as restricting virus release, tetherin also induces activation of NF-κB in response to virus infection (6, 7). HSV-1 has been shown to cause transient NF-κB activation at an early stage of infection, in a replication-independent manner, and this is inhibited by the presence of Vhs in incoming HSV-1 particles (21). Thus, it seems possible that tetherin may be involved in an early NF-κB response to HSV-1, and further studies may shed light on whether Vhs delivered with the virion inhibits NF-κB activity via tetherin depletion.

Herpesviruses have relatively large genomes and can target cellular processes through the activity of more than one gene. Given the importance of evading tetherin, it seems possible that additional HSV-1 proteins could antagonize tetherin function. Indeed, our data show that HSV-1ΔVhs-infected cells still have a somewhat reduced level of tetherin protein and possible altered localization of tetherin at late times postinfection. This implies that other viral factors inhibit tetherin and may act in concert with Vhs. There are several candidate HSV-1 genes that could affect tetherin, such as ICP0 and ICP34.5, which also combat IFN responses (47). As reported in the accompanying paper (48), another possible candidate for antagonizing tetherin is glycoprotein M (gM). We previously demonstrated that gM can internalize viral and cellular membrane proteins to intracellular compartments, and it would be interesting to assess whether gM could also affect tetherin trafficking (49). However, gM is involved in virus assembly by helping to deliver other viral membrane proteins to intracellular assembly compartments (50), and so direct interaction of gM with tetherin could lead to increased concentration of this antiviral factor at HSV-1 assembly sites. Whether gM is a tetherin antagonist and can direct tetherin away from assembly sites, while still mediating viral glycoprotein delivery, and whether other HSV-1 proteins can antagonize tetherin will be interesting questions for future studies.
